# Genome-wide identification of lipoxygenase gene family in cotton and functional characterization in response to abiotic stresses

**DOI:** 10.1186/s12864-018-4985-2

**Published:** 2018-08-09

**Authors:** Muhammad Shaban, Muhammad Mahmood Ahmed, Heng Sun, Abid Ullah, Longfu Zhu

**Affiliations:** National Key Laboratory of Crop Genetic Improvement, Huazhong Agricultural University, Wuhan, Hubei 430070 People’s Republic of China

**Keywords:** Cotton, Lipoxygenase gene family, Promoter, Abiotic stress, Gene expression, Virus-induced gene silencing

## Abstract

**Background:**

Plant lipoxygenase (LOX) genes are members of the non-haeme iron-containing dioxygenase family that catalyze the oxidation of polyunsaturated fatty acids into functionally diverse oxylipins. The LOX family genes have been extensively studied under biotic and abiotic stresses, both in model and non-model plant species; however, information on their roles in cotton is still limited.

**Results:**

A total of 64 putative LOX genes were identified in four cotton species (*Gossypium* (*G. hirsutum*, *G. barbadense*, *G*. *arboreum*, and *G*. *raimondii*)). In the phylogenetic tree, these genes were clustered into three categories (9-LOX, 13-LOX type I, and 13-LOX type II). Segmental duplication of putative LOX genes was observed between homologues from A2 to A_t_ and D5 to D_t_ hinting at allopolyploidy in cultivated tetraploid species (*G. hirsutum* and *G. barbadense)*. The structure and motif composition of *GhLOX* genes appears to be relatively conserved among the subfamilies. Moreover, many *cis*-acting elements related to growth, stresses, and phytohormone signaling were found in the promoter regions of *GhLOX* genes. Gene expression analysis revealed that all *GhLOX* genes were induced in at least two tissues and the majority of *GhLOX* genes were up-regulated in response to heat and salinity stress. Specific expressions of some genes in response to exogenous phytohormones suggest their potential roles in regulating growth and stress responses. In addition, functional characterization of two candidate genes (*GhLOX12* and *GhLOX13*) using virus induced gene silencing (VIGS) approach revealed their increased sensitivity to salinity stress in target gene-silenced cotton. Compared with controls, target gene-silenced plants showed significantly higher chlorophyll degradation, higher H_2_O_2_, malondialdehyde (MDA) and proline accumulation but significantly reduced superoxide dismutase (SOD) activity, suggesting their reduced ability to effectively degrade accumulated reactive oxygen species (ROS).

**Conclusion:**

This genome-wide study provides a systematic analysis of the cotton LOX gene family using bioinformatics tools. Differential expression patterns of cotton LOX genes in different tissues and under various abiotic stress conditions provide insights towards understanding the potential functions of candidate genes. Beyond the findings reported here, our study provides a basis for further uncovering the biological roles of LOX genes in cotton development and adaptation to stress conditions.

**Electronic supplementary material:**

The online version of this article (10.1186/s12864-018-4985-2) contains supplementary material, which is available to authorized users.

## Background

As an important cash crop, cotton is regarded as the backbone in the economy of many developing countries. It not only provides the quality fiber but also cottonseed oil to the world economy [1]. However, during its growth period it encounters various biotic and abiotic stresses. Among the abiotic stresses, salinity causes severe problems in arid and semi-arid regions; it limits crop production by interfering with germination, growth and fertility. Depending on the intensity it causes ion toxicity, water stress, membrane damage, oxidative stress, nutritional imbalances and several cellular and metabolic dysfunctions that can result in the death of plants [2].

The outcome of salinity is increased concentrations of Na^+^ and Cl^−^ ions in plants, which interfere with the normal defense mechanisms against abiotic stresses. In addition, salinity causes hyper-accumulation of reactive oxygen species (ROS), such as overproduction of H_2_O_2_, O_2_^−^, and OH. H_2_O_2_ regulates several physiological processes in plants; however, its overproduction results in several deleterious effects on plants cells. Thus, in order to maintain a balanced level of cellular ROS, plants have evolved mechanisms to detoxify ROS, either enzymatically or non-enzymatically [3]. Generally, cotton is regarded as moderately salt-tolerant crop, but its growth, yield and fiber quality are substantially affected by salt stress [4]. Increasing tolerance to abiotic stresses in order to improve both the yield and fiber quality is one the major challenges for scientists working in this field.

Oxylipins and their derivatives such as jasmonic acid, divinyl ethers, and volatile aldehydes play significant roles during plant responses to biotic and abiotic stresses [5–7]. The synthesis of these oxylipins is catalyzed by LOXs, which are non haeme, iron-containing dioxygenases, ubiquitous in plants and animals [8]. The LOXs catalyze the oxidation of polyunsaturated fatty acids either at carbon atom 9 or at carbon atom 13 and justify their roles as 9-LOX and 13-LOX, respectively. The function of LOX can differ between 9-LOX to 13-LOX due to variations in particular motifs at the specific binding site [9]. The 9-LOX subfamily includes genes which share a high similarity with each other, whereas, 13-LOX subfamily is further categorized into two types according to their similarity and structure. 13-LOX type I genes are more similar to each other (more than 75%) and lack a chloroplast transit peptide; and 13-LOX type II genes show less similarity with each other (up to 35%) and possess a chloroplast transit peptide [10, 11].

The isomers resulting from the functions of 9-LOX or 13-LOX, (9S)-hydroperoxyoctadecadienoic acid (9-HPOD) or (13S)-hydroperoxyoctadecadienoic acid (13-HPOD), further leads to numerous branches of enzymes for the synthesis of different oxylipins [12]. The LOX genes and their derived oxylipins play substantial roles during all stages of plant life such as seed germination, growth, development, and response to stresses [13, 14]. More importantly, the 13-LOX derived oxylipins JA and its precursor (+)-12-oxo-phytodienoic acid (OPDA) have been well characterized for their significant roles during development and response to abiotic stresses [15]. Several reports have highlighted the involvement of JA signaling in salt stress in plants [16–19].

Similarly, the LOX genes have been well documented during plant responses to biotic and abiotic stresses. For example, in pepper, *CaLOX1* plays a crucial role during modulation of abiotic stress responses via rapid scavenging of ROS and activation of defense-related marker genes [20]. In persimmon, *DkLOX3* substantially enhances tolerance to senescence and salt stress by accumulating less O^2−^ and H_2_O_2_ along with the activation of stress-responsive genes [21]. Expression analysis studies also suggest the possible involvement of LOX genes during development and response to biotic and abiotic stress conditions [22–25]. Increased LOX activity is considered to correlate directly with plant salt tolerance mechanisms; such increased LOX activity was observed in many plants, as in rice [26] citrus [27] and tomato [28, 29].

LOX family genes have been comprehensively studied in various plants species, such as in Arabidopsis [30], rice [31] soybean, [32] maize [33] tomato [34], cucumber [35] and others, due to their potential function in various physiological and molecular events. Previous studies have also reported the functions of two LOX genes in cotton during bacterial infection [36, 37]. However, to date, there is no information about genome-wide identification and characterization of LOX gene family members in cotton. With the release of the genomic sequences of four cotton species and the availability of transcriptomic data, it has now become possible to comprehensively characterize and analyze the LOX gene family, which is an essential step to explore the functions of LOX genes in cotton.

In the present study, 64 putative LOX genes were identified during genome-wide screening in four species of cotton. Their phylogenetic relationship, chromosomal distribution, synteny, structure, and expression patterns in various organs, under different abiotic stresses and in response to exogenous phytohormones, were determined. Moreover, we functionally characterized selective LOX genes in response to salt stress using virus induced gene silencing (VIGS). Our study provides insights that will be helpful for further characterization of cotton LOX genes in future.

## Results

### Genome-wide identification of LOX gene family in *Gossypium* spp

To identify LOX family genes in cotton, we initially used the Arabidopsis LOX domain as query to BLAST four cotton genome databases. As a result 21, 18, 11 and 14 putative LOX genes were identified from *G. hirsutum*, *G. barbadense*, *G*. *arboreum* and *G*. *raimondii*, respectively (Additional file 1: Table S1). All of these putative genes fulfilled the criteria of LOX genes as described by Feussner and Wasternack [38]. Multiple sequence alignment (MSA) depicted the presence of representative 38 amino acids motif (His-(X)4-His-(X)4-His-(X)17-His-(X)8-His), highly conserved among all the predicted LOX genes of cotton spp. (Additional file 2: Figure S1). This motif was considered to be essential for enzyme stability and activity; and also to provide binding sites for non-haeme iron-containing dioxygenases [38]. Further properties of these predicted genes, including protein lengths, molecular weights, isoelectric points, functional domains and proposed functionality are provided in Additional file 3: Table S2.

**Fig. 1:**
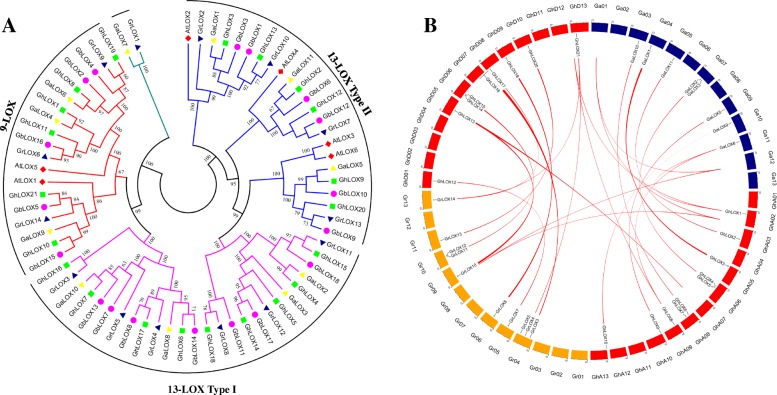
Phylogenetic and synteny analysis of LOX family genes. **a** Neighbor-joining phylogenetic tree was generated with protein sequences of four species of cotton and one model plant Arabidopsis. **b** Syntenic relationships among LOX genes of two diploid (*G. arboreum*, *G*. *raimondii*) and one allotetraploid (*G. hirsutum*) cotton was visualized in a circos plot. The chromosomes of *G. arboreum*, *G*. *raimondii*, and *G. hirsutum* were shaded with blue, yellow, and red colors, respectively

The evolutionary dynamics and syntenic relationships of putative LOX genes among two diploid genomes (*G. arboreum*, *G*. *raimondii*) and sub-genomes in cultivated allotetraploid (*G. hirsutum*) were visualized by generating a circos plot [39] (Fig. 1). The results show that *GaLOX*, *GrLOX* and *GhLOX* genes are distributed among seven chromosomes of A, D and A_t_ subgenomes, respectively, whereas *GhLOX* genes of D_t_ and A_t_ subgenomes were distributed among 7 and 8 chromosomes, respectively. The distribution was uneven, with chromosomes A06, A08, D02, D06, D08 and D09 possessing two *GhLOX* genes, while others (A02, A03, A05, A10, A13, D05 and D13) harbored one *GhLOX* gene (Fig.1b, Additional file 4: Table S3). Moreover, 19 and 29 duplicated gene pairs were identified from A_t_ to A2 and D_t_ to D5, respectively. The genes of each duplicated pair showed greater similarity to each other (Additional file 5: Table S4).

### Phylogenetic analysis of LOX gene family in cotton

To understand the evolutionary relationships of the LOX gene family in the *Gossypium* lineage we constructed a phylogenetic tree for 4 species of cotton and the model plant Arabidopsis using conserved amino acid sequences (Additional file 6: Table S5). As mentioned above, the plant LOX gene family can be grouped into two subfamilies (9-LOX and 13-LOX), according to their positional specificity and catalysis of hydrocarbon backbone [10]. The 13-LOX genes are further categorized into type I and type II, based on their protein structure and similarity [40]. As shown in Fig. 1, all the LOX family genes cluster into three different groups and LOX genes of each species are randomly distributed. Upon inspection of sequence features, we determined that 9-LOX category genes harbor a valine (V) residue (9-LOX motif) at one specific site, responsible for their functional regiospecificity, whereas all the other LOX genes contained phenylalanine (F) (13-LOX motif) and are categorized into 13-LOX subfamily, with the exception of *GaLOX7* and *GrLOX1* (Additional file 7: Figure S2). Both of these genes possess a leucine (L) residue at the specific site instead of phenylalanine or valine, and form a distinct branch in the phylogenetic tree. There are only few reports about the categorization of such kinds of LOX genes in plants [22].

Subcellular localization of LOX proteins was predicted using three different publicly available programs. The results revealed that 13-LOX type I proteins are preferentially found in the cytoplasm, while type II 13-LOXs are in chloroplast (Additional file 8: Table S6). More investigation showed that cotton 9-LOX genes formed association with Arabidopsis 9-LOX genes *AtLOX1* and *AtLOX5*, reported to play roles in protection of plants against various biotic and abiotic stresses through the synthesis of diverse oxylipins [41, 42]. The 13-LOX subfamily clustered with *AtLOX2*, *AtLOX3*, *AtLOX4*, and *AtLOX6*, whose roles are well established in growth, development, jasmonic acid biosynthesis, and defense signaling against stress conditions [43–45]. More often, it is considered that genes with similar functions cluster together; the distribution of cotton LOX genes in different clusters might explain their probable functions.

### Structural and motif analysis of *GhLOX* genes

**Fig. 2:**
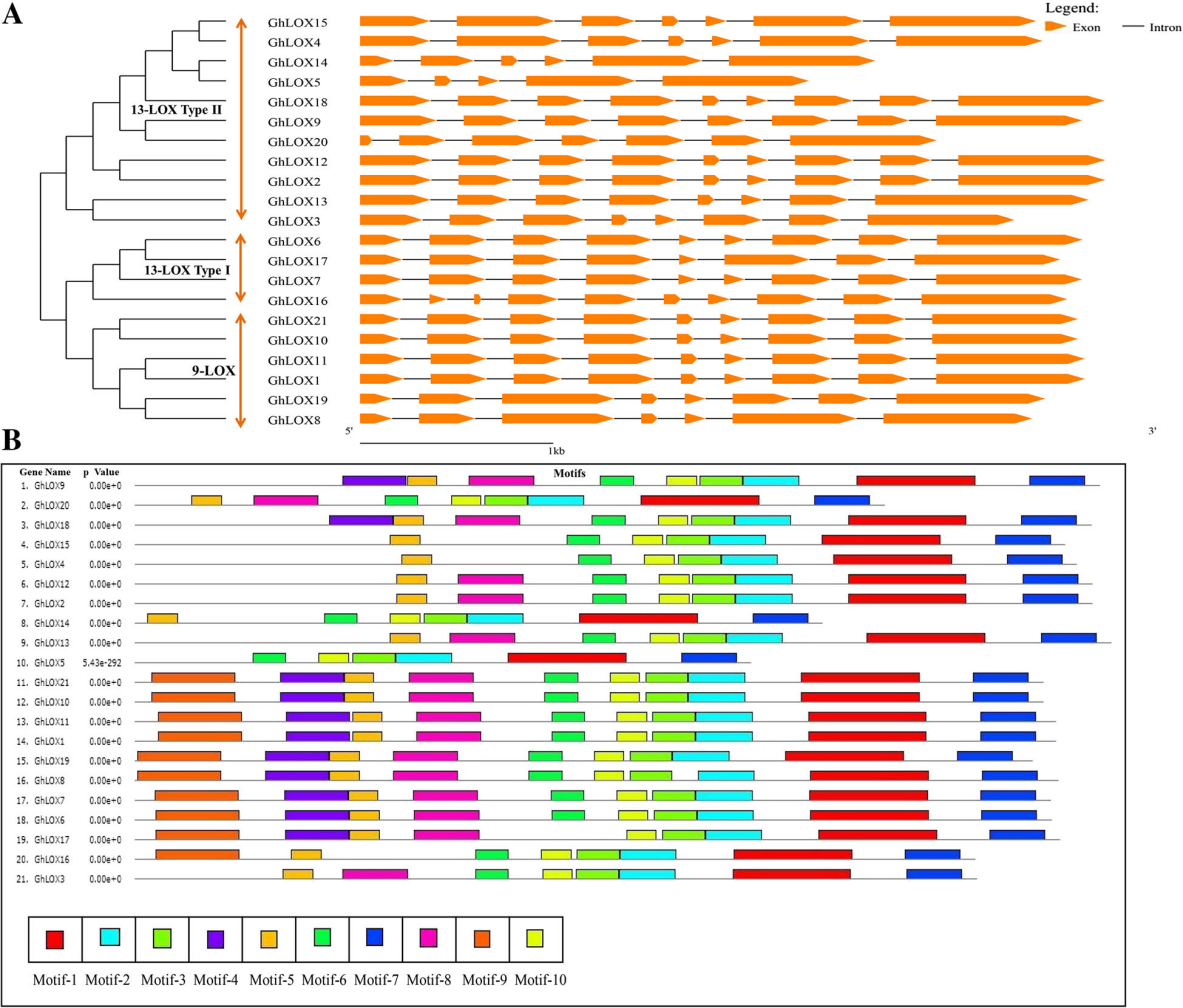
Structural and motif analysis of *GhLOX* genes. **a** Structural analysis. Neighbor-joining phylogenetic tree was constructed among *GhLOX* genes with MEGA7. The subfamilies were marked correspondingly. The sizes of exons are relative to their sequence length. **b** Motif analysis. Ten distinct motifs were identified with MEME suite and each motif was represented with different color

To gain further knowledge about the structure and motif composition of *GhLOX* genes, we constructed a separate phylogenetic tree and analyzed their structural characteristics through the Gene Structure Display web portal. Based on the results of phylogenetic tree, *GhLOX* genes were divided into three subfamilies (9-LOX, 13-LOX type I and type II). The number of introns and length of exons among most of the *GhLOX* genes either 9-LOX or 13-LOX were found to be similar (Fig. 2a). However, we observed variation in gene structure of some 13-LOX type II genes. The minimum number of introns (4 introns) was found in *GhLOX5* and maximum number in *GhLOX16* (9 introns).

To explore conserved motifs within *GhLOX* subfamilies, we used MEME Suite (*Ver*. 4.12.0) to predict distinct motifs shared by the *GhLOX* family. We identified 10 distinct motifs. Most of these motifs encoded a LOX domain (Fig. 2b and Additional file 9: Table S7). As expected, most of the structurally similar members shared common motifs within the same subfamily, suggesting their similar functions. Upon closer inspection, it was found that 5 motifs (motifs 1–3, 7, 10) were shared by all the *GhLOX* genes. However, it is noteworthy that some of the specific motifs were absent in specific subfamilies. For example, motif 9 was absent in all the members of 13-LOX subfamily type II, and motif 4 was absent in most of the members of this subfamily. However, whether the presence or absence of these specific motifs confers unique functional roles to LOX genes need further research. In any case, the structure and motif conservation within subfamilies may additionally support the results of phylogenetic analysis. Alternatively, variations in motif composition between subfamilies might be explained by their functional diversification.

### *Cis*- elements analysis of *GhLOX* genes

**Fig. 3:**
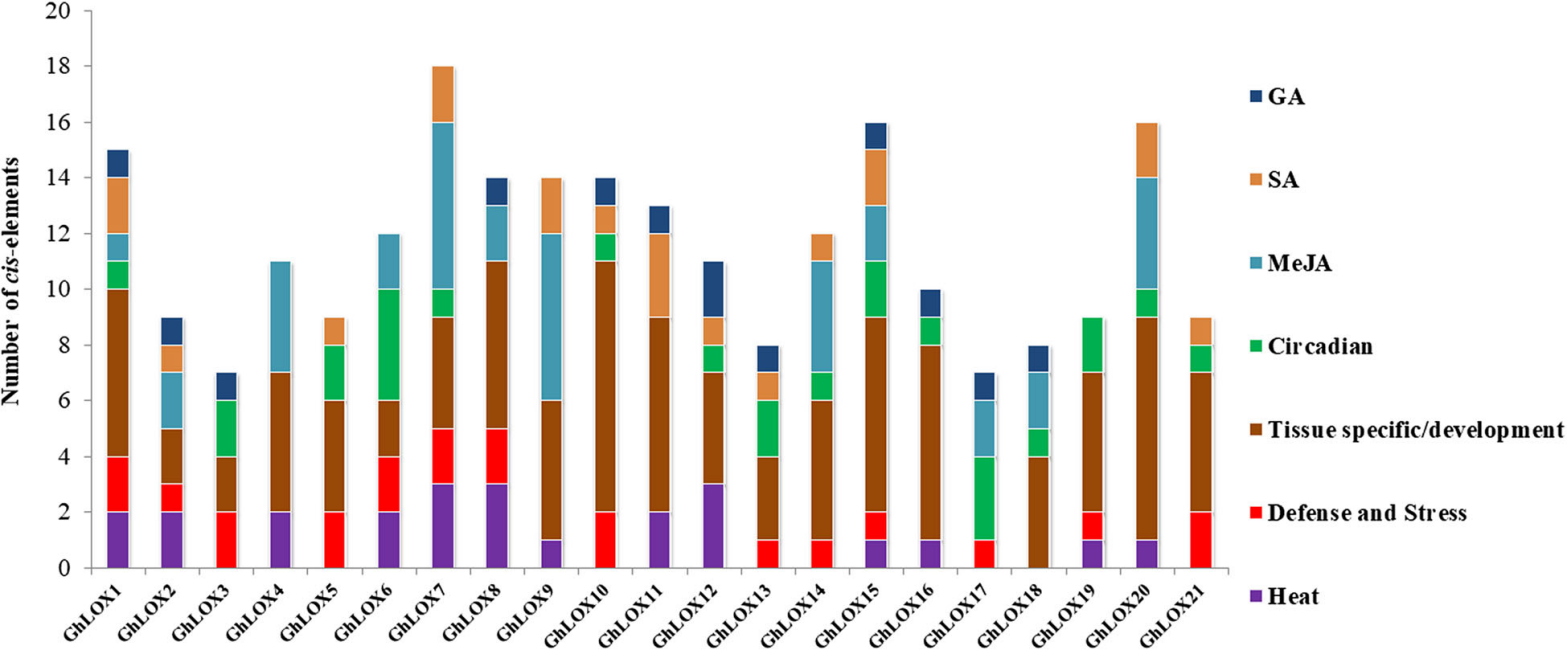
Promoter analysis of *GhLOX* genes. The numbers of different *cis*-elements were presented in the form of bar graphs; similar *cis*-elements were shown with same colors

*Cis*-elements in promoters play vital roles in regulating the expression of genes. These are in regions of non-coding DNA, usually found upstream of transcriptional start sites. In most of cases, gene expression depends on the presence or absence of these elements [46]. To analyze *cis*-elements potentially involved in the regulation of *GhLOX* genes, we selected a 1.5 kb 5′ flanking region upstream from the start codon of each *GhLOX* gene. The PLANTCARE database identified several stress, phytohormone, developmental and light responsive *cis*-regulatory elements in their promoter regions (Fig. 3 and Additional file 10: Table S8). Out of 21 *GhLOX* genes, 20 possessed tissue-specific or development-responsive elements, with 16 enriched in circadian-responsive elements, 15 contained ARE (anaerobic induction-responsive element), 14 had TC-rich repeats (defense or stress-related), 13 harbored the HSE (heat stress element), and 8 genes possessed MBS (MYB-binding sites), responsible for abiotic stress responses. All the *GhLOX* genes contained large numbers of light-responsive elements. Moreover, many of the *GhLOX* genes also harbored the TCA, CGTCA/TGACG, GARE/P-box encoding *cis*-acting elements in their promoter regions, responsible for SA (salicylic acid), JA (jasmonic acid), and GA (gibberellic acid) signaling, respectively.

### Tissue specific expression patterns of *GhLOX* genes

**Fig. 4:**
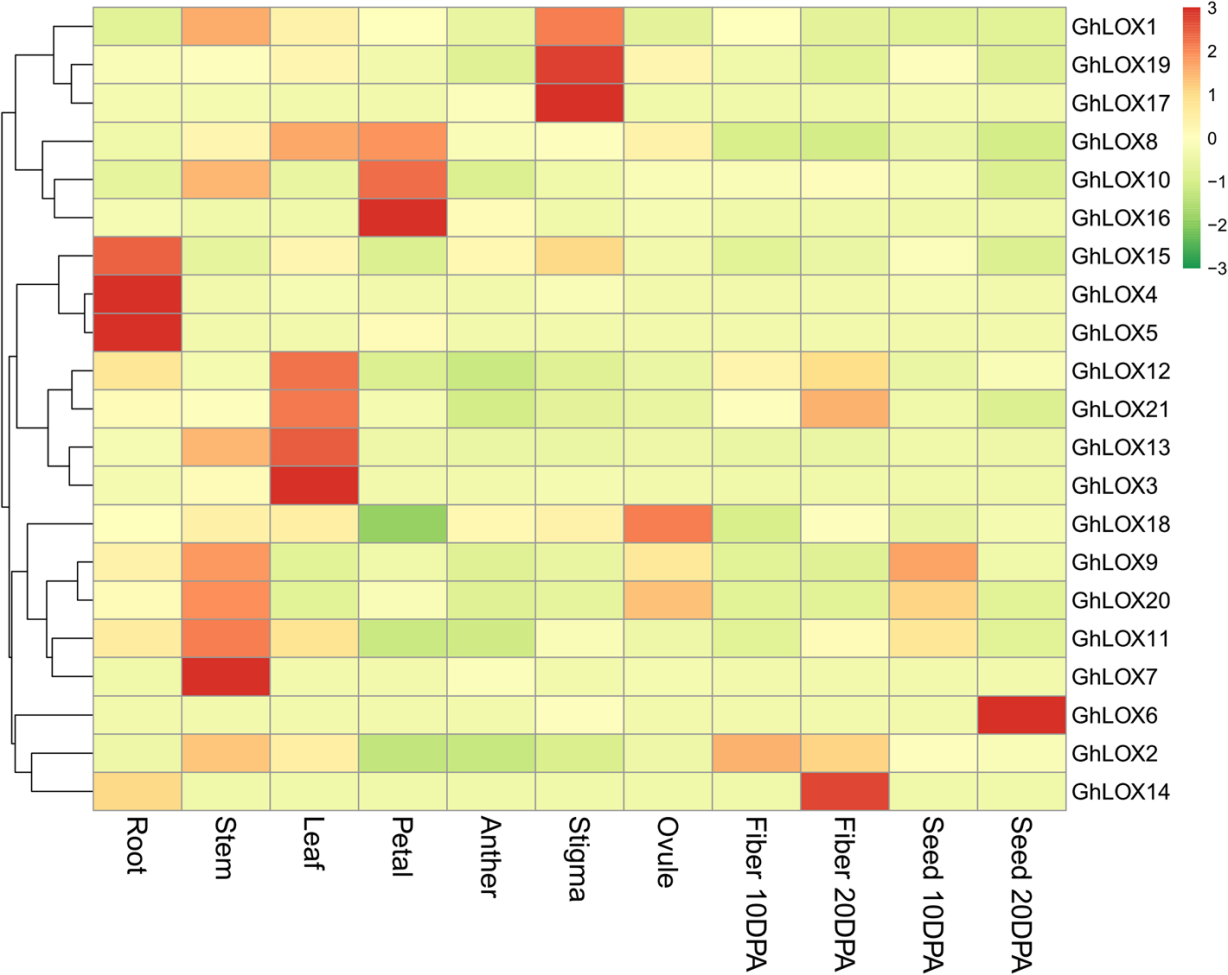
Expression pattern of *GhLOX* genes in different tissues of *G. hirsutum*. The transcriptomic data related to tissue expression were accessed from NCBI and the *pheatmap* package was used for the generation of heatmaps

To determine the expression of *GhLOX* genes in various tissues of *G. hirsutum* (root, stem, leaves, flowers, fiber and seeds), we investigated the transcriptomic data provided by Zhang et al. [47]. The expression analysis showed that transcripts of all the *GhLOX* genes were detected in at least two tissues (Fig. 4 and Additional file 11: Table S9). However, 3 genes (*GhLOX10*, *GhLOX19*, and *GhLOX21*) were expressed in all the tested tissues [Fragments per kilobase of transcript per million mapped reads (FPKM ≥ 1)]. In addition, 6 *GhLOX* genes (*GhLOX2*, *GhLOX9–10*, *GhLOX13*, and *GhLOX20*–*21*) were highly expressed in stem, with highest expression noted for *GhLOX20* (FPKM ≥ 46) and 7 genes (*GhLOX2*–*3*, *GhLOX8*, *GhLOX12*–*13*, *GhLOX19* and *GhLOX21*) strongly induced in leaves, with highest expression observed for *GhLOX21* (FPKM ≥ 61). Based on the expression pattern of *GhLOX* genes in cotton and the reported roles of their orthologues in Arabidopsis, we can assume the probable functions of these genes. *GhLOX21* is highly expressed in leaves and also had strong expression in roots (FPKM ≥ 23), and its ortholog is *AtLOX1*, reported to be involved in lateral root development [41]. Similarly, along with higher expression of *GhLOX9* and *GhLOX20* in stem, both of these genes are also strongly induced in seeds at 10 DPA (FPKM ≥ 30), and more strongly than other *GhLOX* genes. Their ortholog is *AtLOX3*, reported to regulate seed development [30]. This suggests that *GhLOX21*, *GhLOX9* and *GhLOX20* perform similar functions in cotton to *AtLOX1* and *AtLOX3* in Arabidopsis. However, this expression trend was not observed in all the *GhLOX* genes, suggesting that some LOX genes may adopt different physiological functions across species.

### Expression analysis of *GhLOX* genes under different abiotic stresses

**Fig. 5:**
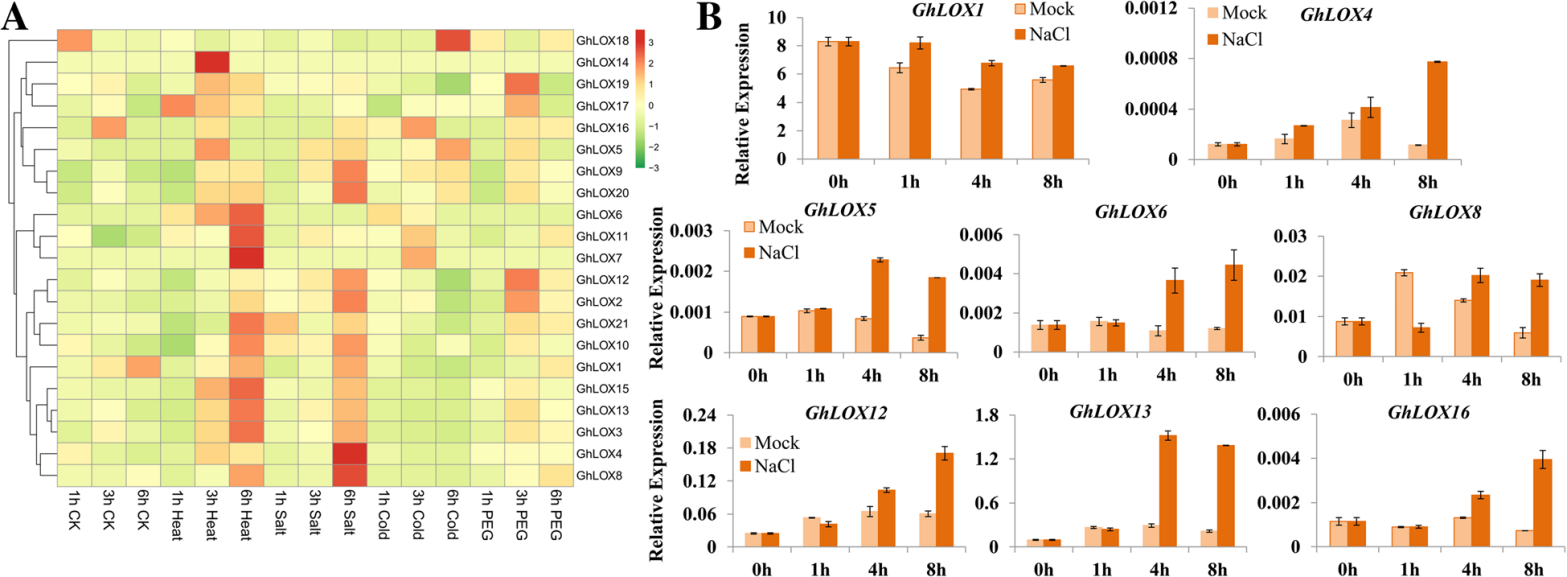
Expression profile of *GhLOX* genes in response to different abiotic stresses. **a** Abiotic stress related transcriptomic data were obtained from NCBI and its normalization and visualization was performed using the *pheatmap* package. **b** The relative expression of selected *GhLOX* genes under 200 mM salt stress was determined by qRT-PCR. The cotton *UBQ7* gene was used as internal control. Error bars denotes the standard deviation calculated from three independent experiments

Considering potential roles of LOX genes and their metabolites in various plant species against biotic and abiotic stresses, we investigated the transcript abundance of *GhLOX* genes in response to heat, salt, polyethylene glycol (PEG) and cold, using transcriptomic data of *G. hirsutum* provided by Zhang et al. [47]. The results show that all the *GhLOX* genes were differentially expressed under one or more stresses (Fig. 5a). Comparing four stress factors, more *GhLOX* genes showed altered expression in response to heat and salinity than to osmotic (PEG) and cold stresses. Interestingly, most of the *GhLOX* genes which showed altered expression in response to heat, also expressed differentially to salinity stress, suggesting the existence of similar biological processes affected by *GhLOX* genes in modulating these stress responses in cotton. Eight *GhLOX* genes were also induced significantly (treatment RPKM/control RPKM ≥ 2) under heat stress, with highest fold change observed for *GhLOX7*. In response to salinity stress, nine *GhLOX* genes showed increased expression (treatment RPKM/control RPKM ≥ 1.5) over the 3 h to 6 h time period. Some genes demonstrated interesting expression profiles. For example *GhLOX18* was only induced under 6 h cold stress, and *GhLOX19* under 3 h PEG stress (treatment RPKM/control RPKM ≥ 2), suggesting potential functions for these genes in cold and osmotic stress in cotton. The expression profiles of selected genes, assessed through quantitative real time polymerase chain reaction (qRT-PCR), further verified the results of the transcriptomic datasets. The results show that most of the selected *GhLOX* genes were induced after 4 h of salt treatment and maintained high levels of expression up to 8 h (Fig. 5b).

### Expression patterns of *GhLOX* genes under different exogenous phytohormone treatments

**Fig. 6:**
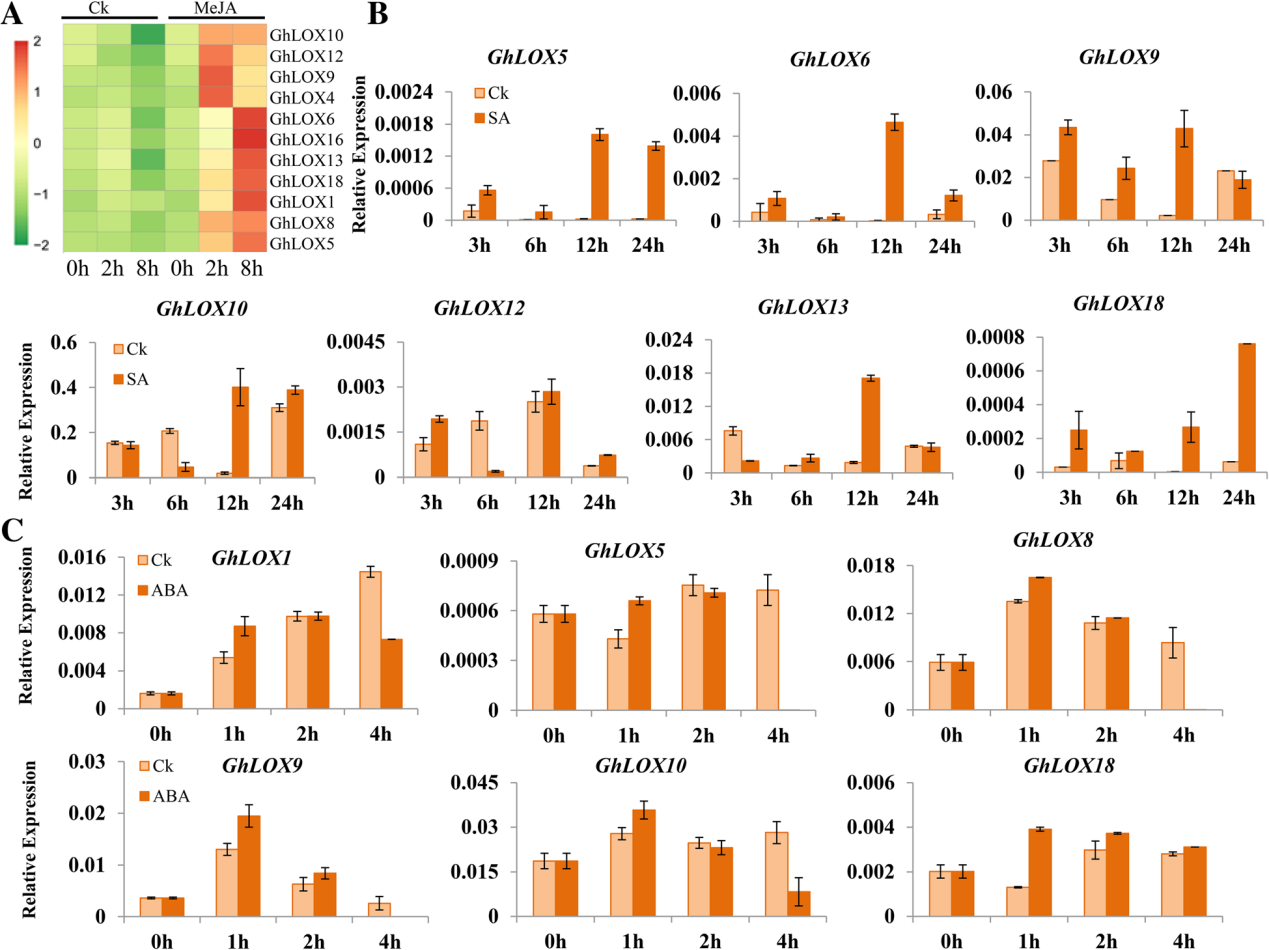
Expression analysis of *GhLOX* genes following treatment with exogenous phytohormones. **a** Expression levels of *GhLOX* genes in response to 100 μM MeJA treatment were determined by qRT-PCR analysis and visualized as heat map using the *pheatmap* package. **b** Responses of selective *GhLOX* genes under 1 mM SA were measured by qRT-PCR. **c** Expression analysis of selective *GhLOX* genes under 0.5 μM ABA treatment was performed by qRT-PCR. The cotton *UBQ7* gene was used as internal control. Error bars denotes the standard deviation calculated from three independent experiments

Phytohormones play important roles in alleviating adverse abiotic stresses in plants, along with many other biological functions [17]. To elucidate the potential functions of *GhLOX* genes in response to exogenous phytohormones, we determined the expression pattern of *GhLOX* genes by qRT-PCR. From the homologous genes pairs, we selected one pair and designed gene-specific primers. The expression of all the *GhLOX* genes was significantly induced in response to MeJA (methyl jasmonate) treatment compared to controls (Fig. 6a). Differential expression of some selected genes was observed following SA treatment. Most genes exhibited higher expression by 12 h after treatment (Fig. 6b). Interestingly, following ABA (abscisic acid) treatment most of the selected *GhLOX* genes were highly induced during the early time periods and reduced during the later time periods (Fig. 6c).

### Silencing of *GhLOX12* and *GhLOX13* compromises cotton tolerance to salt stress

**Fig. 7:**
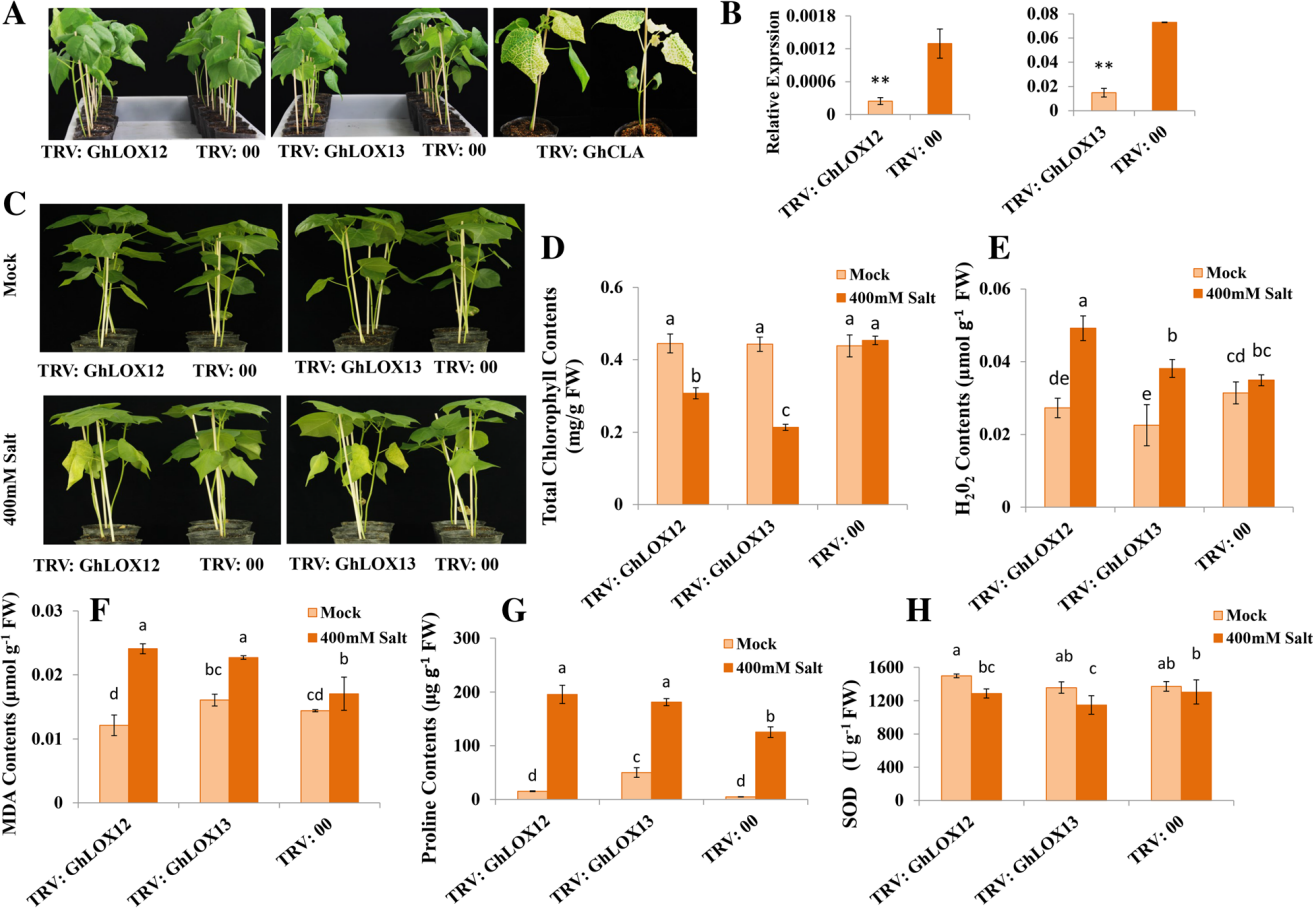
Silencing of *GhLOX12*, *GhLOX13* compromises tolerance to salt stress in target gene-silenced cotton. **a** The target gene-silenced, control and positive control plants before salt stress. **b** Relative expression levels of target gene-silenced and control plants. **c** Phenotype (**d**) total chlorophyll contents, **e** H_2_O_2_ contents (**f**) MDA contents (**g**) proline contents and (**h**) SOD activity between target gene-silenced and control plants under mock and salt stress treatment. Experiments were repeated three times, the values represent the means and the error bars show standard deviation and asterisks represents the significant difference at *P ≥ 0.05, **P ≥ 0.01, Student’s t-test, different letters denote the significant difference (*P* ≥ 0.05) between each other according to LSD test

Based on the integration of promoter analysis and differential expression patterns under different abiotic stresses we hypothesized that *GhLOX* genes are potentially important in the regulation of stress responses. Further salt-induced expression patterns of selective candidate genes inspired us to investigate their functional importance in cotton. To verify our hypothesis, we adopted a VIGS approach to knock down the expression of two LOX genes, *GhLOX12* and *GhLOX13* using TRV vectors, (TRV: GhLOX12) and (TRV: GhLOX13) respectively. The results revealed that *GhLOX12* and *GhLOX13* were successfully knocked down in cotton 2 weeks after VIGS operation (Fig. 7b). TRV: GhCLA was used as positive control. Two weeks following VIGS, when positive control plants changed phenotype (Fig. 7a), qRT-PCR was employed to determine the expression levels in the leaves of TRV: GhLOX12, TRV: GhLOX13 and TRV: 00 control plants. The results show that the transcript levels of both *GhLOX12* and *GhLOX13* were significantly reduced following 2 weeks of VIGS.

Half of the target gene-silenced (TRV: GhLOX12, TRV: GhLOX13) and control plants (TRV: 00) were then irrigated with water as mock and half with 400 mM NaCl as salt stress treatment. After 10 days, there was no phenotypic difference between control and target gene-silenced plants in response to water treatment. However, target gene-silenced plants displayed severe wilting and yellowing of leaves compared to control plants (TRV: 00) under salt stress, consistent with an observed decrease in chlorophyll contents (Fig. 7c and d). Abiotic stresses in plants elevate the level of ROS (mainly of O_2_− and H_2_O_2_); which ultimately affect plant metabolism, signal transduction and damages the plant cells. To determine the possible roles of ROS in salt stressed plants; we measured the H_2_O_2_ contents in target gene-silenced and control plants under salinity and mock conditions. The H_2_O_2_ contents were induced significantly in the leaves of both target gene-silenced plants as compared with control plants under salt stress (Fig. 7e). The malondialdehyde (MDA) level in plants is considered as a marker of abiotic stress response, reflecting membrane damage or injury. Similar to H_2_O_2_, MDA levels was significantly higher in target gene-silenced plants as compared to control plants under salt stress (Fig. 7f). Interestingly, proline, which is a non-toxic osmolyte and plays important roles in osmotic adjustment and turgidity of cells under stress conditions, also increased dramatically following salinity treatment both in target gene-silenced and control plants, however there was significant difference between them (Fig. 7g). Furthermore, to explore the function of target gene-silencing in the modulation of antioxidant enzymes, we measured the superoxide dismutase (SOD) activity of salt stressed and mock treated plants. As expected, SOD activity decreased under salt treatment in both target gene-silenced and control plants as compared to mock. The target gene-silenced plants displayed a significantly reduced SOD activity as compared to control plants under salt stress (Fig. 7h).

## Discussion

The LOX gene family has been extensively studied in model and non-model plant species for their potential functions in growth, development, and responses to biotic and abiotic stresses. However, previously such efforts were not directed towards understanding the cotton LOX gene family. By using Arabidopsis LOX genes as query, we searched the four cotton genomes, and identified 64 homologous LOX genes. In *G. hirsutum*, we identified 21 genes, which is a comparatively large gene family relative to the other three species of cotton. Additionally, the numbers of *GhLOX* genes were not twice that of two diploid cotton species, presumably due to genomic variations occurring during paleopolyploidization in the *Gossypium* lineage, which ultimately led to the evolution of cultivated tetraploid cotton. Of 13 chromosomes, all the *GaLOX* and *GrLOX* genes were distributed among 7 chromosomes while *GhLOX* genes were found on 7 chromosomes of the D_t_ subgenome and on 8 chromosomes of the A_t_ subgenome. Further systematic analysis is needed to unfold insights into LOX gene family evolution in cotton.

During phylogenetic analysis, the putative LOX genes were grouped into three subfamilies based on their protein structure and similarity. Gene prediction analysis showed that all the 13-LOX type II genes encode chloroplastic proteins, while most of 9-LOX and 13-LOX type I proteins are preferentially localized in the cytoplasm. Multiple sequence alignment of conserved amino acids residues revealed the presence of conserved motifs corresponding to these categories, suggesting the functional conservation among these subfamilies. Gene structure analysis showed that most of the 9-LOX and 13-LOX type I *GhLOX* genes shared similar structures. However, we observed some variation in the structure of 13-LOX type II genes, which might be explained by their functional diversification. To gain further insights into the regulation of *GhLOX* genes under changing environmental conditions, we investigated the *cis*-regulatory elements inside their promoter regions. Mainly five types of *cis*-acting elements were identified, including defense/stress-related (biotic and abiotic), tissue specific/development-related, hormones-responsive, light-responsive and circadian-related. Such a wide spectrum of *cis*-acting elements is consistent with the published reports about the multifunctional roles of LOX genes in plants [13].

The expression profile of *GhLOX* genes in different tissues revealed that most of the genes are expressed in vegetative tissues, suggesting that *GhLOX* genes play important roles in the development of vegetative tissues in cotton. In addition, significantly higher expression of some genes in only specific tissues, such as *GhLOX14* in fiber at 20 DPA, *GhLOX6* in seed at 20 DPA, and *GhLOX17* and *GhLOX19* in stigma suggests likely functional importance in these organs.

The expression pattern of *GhLOX* genes obtained from transcriptomic data under abiotic stresses revealed that most of the genes are associated with heat and salt stress simultaneously and there might be a similar mechanism of tolerance prevailing in cotton under these stresses. However, we observed different expression pattern of some genes during heat and salt stress, such as *GhLOX6–7* and *GhLOX14*, which were only responsive to heat stress, and no changes in expression were observed for salt stress. Moreover, the results obtained in expression analysis of selective LOX genes under salt stress through qRT-PCR were in agreement with the transcriptomic dataset. The observed significant induction of *GhLOX* genes in response to phytohormones further supports their probable involvement in modulating stress conditions. More intriguingly, the comparative analysis of significantly induced genes under heat and salt stress and their promoter sequences might help explain the reasons for their responsiveness to these stresses. For example, *GhLOX12* harbored 3 HSE, 1 MBS, 1 LTR (low temperature-responsive), 2 ARE and 2 TC-rich repeat elements.

Salinity is an increasing problem for world agriculture; it affects more than 20% of irrigated areas [48]. Cotton, a relatively salt tolerant crop is also nevertheless adversely affected by salinity during germination and seedling stages of development. Losses can reach 50% of seed yield, if the salinity is above a threshold level [49]. Our genome-wide screening of LOX genes in cotton, their phylogeny, promoter and expression analysis suggests their potential in modulating salinity tolerance. VIGS silencing of two LOX genes, *GhLOX12* and *GhLOX13* led to a compromised plant tolerance to salt stress, confirming their functional importance.

To explore the possible mechanism of their sensitivity, we analyzed some physiological parameters including total chlorophyll contents, accumulation of H_2_O_2_, MDA, proline, and activity of SOD both under salt stress and mock treatment. The results showed that silencing of the target LOX genes in cotton significantly effects the growth and physiology under salinity. Some physiological traits were particularly adversely affected by silencing of *GhLOX12* and *GhLOX13*. Chlorophyll contents and activity of antioxidant enzyme (SOD) were significantly decreased in LOX gene-silenced plants, whereas H_2_O_2_, MDA, and proline contents were found to be increased significantly under salt stress. Abiotic stresses often result in overproduction of ROS, which cause the lipid peroxidation, interfering with normal physiological processes and ultimately leading to programmed cell death. The scavenging of ROS is an important mechanism of tolerance against salt and osmotic stresses [50]. The higher accumulation of H_2_O_2_, MDA and reduced SOD activity after silencing of *GhLOX12* and *GhLOX13* suggests the reduced ability of silenced plants to properly scavenge ROS that leads to membrane damages and chlorophyll reduction. These results are consistent with previous studies where LOX genes have been implicated in salt stress tolerance by modulating ROS [20, 21]. The increased contents of proline in silenced plants following salinity treatment might be explained by the need of a substitution process to effectively detoxify elevated ROS. Substitution processes may be necessary when the activities of antioxidant enzymes such as SOD are decreased in silenced plants [51].

## Conclusion

The decoding of cotton genomes allowed us to perform a genome-wide screening for LOX family genes. Through a systematic approach, comprising analysis of gene structure, phylogeny, motif composition, promoter structure, and expression patterns in diverse tissues, in response to signaling molecules, and under various abiotic stress factors, we were able to comprehensively characterize the LOX family genes in cotton. Results suggest the conservation and diversity of sequence features and functions among LOX family genes in different species of cotton. Moreover, the functional characterization of two LOX genes (*GhLOX12* and *GhLOX13*) through a VIGS approach supported their role in salinity tolerance, possibly via regulating ROS. This comprehensive analysis provides a foundation for future functional studies to decipher the functional roles of all LOX genes in cotton and potentially other species. The data presented here may help in the selection of appropriate candidate genes for further functional characterization related to a specific aspect of cotton development or under stress conditions.

## Methods

### Isolation and sequence retrieval of LOX gene family from four species of cotton

The genome assemblies of four cotton species (*G. arboreum*, *G. raimondii*, *G. barbadense* and *G. hirsutum*) were retrieved from cottongen (https://www.cottongen.org). The sequences of Arabidopsis were accessed from TAIR website (https://www.Arabidopsis.org/). By employing the BLAST+ program [52] Arabidopsis LOX genes were used as query to identify putative LOX genes in four cotton genomes. All the putative LOX genes were checked for the presence of LOX domain through pfam database (http://pfam.sanger.ac.uk/) [53], NCBI Conserved Domain Database (http://www.ncbi.nlm.nih.gov/Structure/cdd/wrpsb.cgi) [54], SMART (http://smart.embl-heidelberg.de/) [55] and InterProScan programs (http://www.ebi.ac.uk/Tools/InterProScan/) [56]. Moreover, KEGG pathway numbers were determined by extracting and mapping gene ontology (GO) terms through Blast2GO program [57, 58]. Several of the redundant sequences were removed for not having the complete domain or shortness.

### Phylogenetic and subcellular localization analysis

Multiple sequence alignments of selected LOX genes were performed through ClustalX software (*ver*. 1.83) and conserved motifs were manually screened and highlighted using online shading tool (http://www.bioinformatics.org/sms2/color_align_cons.html). Further, conserved amino acid sequences were employed to Molecular Evolutionary Genetics Analysis (MEGA 7) software for construction of phylogenetic tree, with Neighbor Joining method having 1000 bootstrap values [59]. Subcellular localization prediction of LOX genes were carried out using three publically available web tools ProtComp 9.0 (http://linux1.softberry.com/berry.phtml), Target P1.1 (http://www.cbs.dtu.dk/services/TargetP) and PSORT (http://wolfpsort.org/) [60].

### Genes structure, conserved motifs and promoter analysis

The organization of exon/intron boundaries was carried out online via accessing Gene Structure Display Server (GSDS, V.2) (http://gsds.cbi.pku.edu.cn/) [61]. MEME Suite (http://meme-suite.org/index.html) [62] was employed for identification of conserved motifs, with default setting except changes in number of motifs (10) and width (upto 200 residues). The structural annotation of distinct motifs was carried out using Pfam database [53]. The potential *cis*-elements in the promoter sequences of *GhLOX* genes were identified using PlantCARE program (http://bioinformatics.psb.ugent.be/webtools/plantcare/html/).

### Plant material and treatment for expression analysis

The transcriptomic data of *G. hirsutum* ‘TM1’ related to tissue expression and in response to various abiotic stress factors were downloaded from NCBI (https://www.ncbi.nlm.nih.gov/sra/?term=PRJNA248163). The resulting expression data were then used for generation of heatmaps by *pheatmap* package (pheatmap: R package version 1.0.8, https://CRAN.R-project.org/package=pheatmap).

To check the expression profile of *GhLOX* genes by qRT-PCR, *G. hirsutum cv.* YZ1 seedlings were grown in Hoagland solution under controlled conditions (25 °C,16 h light/8 h dark cycle). After 4 weeks, plants were subjected to Hoagland solution containing water as mock treatment or 200 mM NaCl as salt stress treatment, or 100 μM MeJA, 1 mM SA and 0.5 μM ABA as hormones treatments. Leaf samples were taken at desired time points and stored for subsequent RNA extraction.

### Vector construction and procedure for VIGS in cotton

Three hundred seventy-seven bp and 400 bp fragments from the coding DNA sequence of *GhLOX12* and *GhLOX13* were amplified from *G. hirsutum cv*. Y668 cDNA, respectively and subsequently introduced into the TRV: 00 plasmid. DNA was digested with restriction enzymes BamHI and KpnI to generate the TRV: GhLOX12 and TRV: GhLOX13. The TRV: GhLOX12, TRV: GhLOX13 and TRV1 construct were inserted into *A. tumefaciens* strain GV3101 by electroporation. We followed the same procedure for VIGS in cotton as mentioned previously by Gao et al. [63]. The primers used for vectors construction are mentioned in Additional file 12: Table S10.

### Plant material and salt tolerance assays for functional analysis of *GhLOX* genes

*G. hirsutum cv*. Y668 seedlings were grown in small pots of soil under controlled condition (25 °C, 16 h/8 h light/dark period) in incubator. After verifying the VIGS efficiency through qRT-PCR, the roots of both control and target gene-silenced plants were irrigated with water as mock treatments and 400 mM NaCl as salt stress up to 10 days. The VIGS experiments were repeated with three replicates, and 40 plants were used during each replication. Half of the plants were treated with NaCl for salt stress tolerance assay and half of the plants were treated with water as mock.

### Determination of salt stress-related physiological parameters

Total chlorophyll contents were determined from 0.1 g cotton leaf tissues and 80% acetone was used as extraction buffer. The absorbance was measured at 663 nm and 645 nm using a Multimode plate reader. For calculation of total chlorophyll contents, we followed the method described by Sun et al. [64]. For quantification of H_2_O_2_ contents, 0.1 g samples of cotton leaves were prepared and followed the procedure as described in H_2_O_2_ Quantification Assay Kit (Sangon Biotech, China). The measurement of proline contents and SOD enzyme activity from stressed and control plants were as described by Yu et al. [65]. To assess the lipid peroxidation, we measured the MDA contents following the procedure of Hodges et al. [66].

### qRT-PCR analysis

To investigate gene expression patterns, total RNA was extracted from leaves of *G. hirsutum* following the protocol of Tu et al. [67]. RNA was reverse transcribed into cDNA using M-mlv reverse transcript system (Promega, USA). qRT-PCR was performed on an ABI 7500 real time PCR system (Applied Biosystem, Foster City, CA, USA) as described by Xu et al. [68]. The fold changes were calculated using the comparative CT method (2^-∆∆Ct^) and *GhUBQ7* was amplified as an internal control. Gene-specific primers were designed based on cDNA sequences. All the primers used in this study were designed using the software primer premier 5 and listed in Additional file 12: Table S10.

## Additional files

Additional file 1: **Table S1.** Nomenclature of LOX gene family in four species of cotton. (XLSX 11 kb)

Additional file 2: **Figure S1.** Multiple sequence alignment of LOX gene family in four species of cotton. Circled boxes show the representative 38 amino acids motif in G. arboreum (A), G. raimondii (B), G. barbadense (C), and G. hirsutum (D). (PDF 653 kb)

Additional file 3: **Table S2.** Properties of LOX gene family in four species of cotton. (XLSX 13 kb)

Additional file 4: **Table S3.** Chromosomal location of LOX gene family in four species of cotton. (XLSX 11 kb)

Additional file 5: **Table S4.** Similarity index of duplicated gene pairs between At to A2 and Dt to D5 subgenomes. (XLSX 9 kb)

Additional file 6: **Table S5.** Protein sequences used for construction of phylogenetic tree. (XLSX 31 kb)

Additional file 7: **Figure S2.** The 9-LOX and 13-LOX specific motif among LOX gene family in four species of cotton. The encircled column represents the specific motif in G. arboreum (A), G. raimondii (B), G. barbadense (C), and G. hirsutum (D). (PDF 478 kb)

Additional file 8: **Table S6.** Subcellular localization of LOX gene family in four species of cotton. (XLSX 47 kb)

Additional file 9: **Table S7.** The 10 distinct motifs found in GhLOX genes. (XLSX 10 kb)

Additional file 10: **Table S8.** cis-acting elements in the promoters of GhLOX genes. The values represent the numbers of specific cis-acting elements in each GhLOX genes. (XLSX 11 kb)

Additional file 11: **Table S9.** GhLOX genes tissue expression profile. The data represents the FPKM values. (XLSX 12 kb)

Additional file 12: **Table S10.** Primers used in this Study. (DOCX 16 kb)

## Abbreviations

13-HPOD: (13S)-hydroperoxyoctadecadienoic acid
9-HPOD: (9S)-hydroperoxyoctadecadienoic acid
ABA: Abscisic acid
FPKM: Fragments per kilobase of transcript per million mapped reads
GAs: Gibberellins
JA: Jasmonic acid
LOX: Lipoxygenase
MeJA: Methyl jasmonate
OPDA: (+)-12-oxo-phytodienoic acid
qRT-PCR: Quantitative Real time Polymerase Chain Reaction
ROS: Reactive Oxygen Species
SA: Salicylic acid
SOD: Superoxide dismutase
VIGS: Virus induced gene silencing
