# Supplementary material for: Genome-wide identification of lipoxygenase gene family in cotton and functional characterization in response to abiotic stresses
Source: BMC Genomics. 2018 Aug 9;19:599. doi: 10.1186/s12864-018-4985-2 (PMC6085620; doi:10.1186/s12864-018-4985-2)

A representative 38 amino acids motif

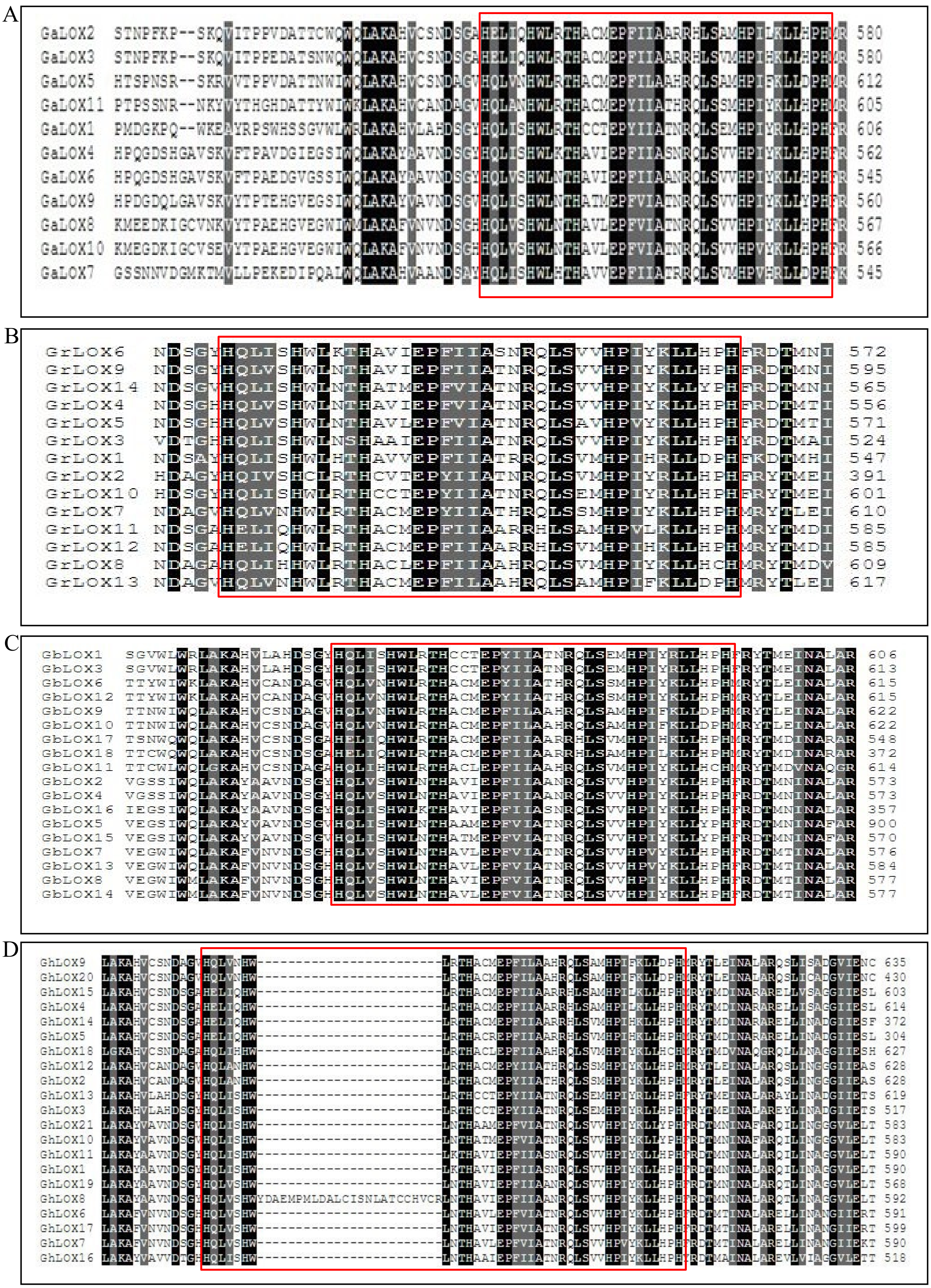

Supplement: Supplementary file 2 — Figure S1. Multiple sequence alignment of LOX gene family in four species of cotton. Circled boxes show the representative 38 amino acids motif in G. arboreum (A), G. raimondii (B), G. barbadense (C), and G. hirsutum (D). (PDF 653 kb) [file 12864_2018_4985_MOESM2_ESM.pdf]
