# Supplementary material for: Genome-wide identification of lipoxygenase gene family in cotton and functional characterization in response to abiotic stresses
Source: BMC Genomics. 2018 Aug 9;19:599. doi: 10.1186/s12864-018-4985-2 (PMC6085620; doi:10.1186/s12864-018-4985-2)
Supplement: Supplementary file 7 — Figure S2. The 9-LOX and 13-LOX specific motif among LOX gene family in four species of cotton. The encircled column represents the specific motif in G. arboreum (A), G. raimondii (B), G. barbadense (C), and G. hirsutum (D). (PDF 478 kb) [file 12864_2018_4985_MOESM7_ESM.pdf]

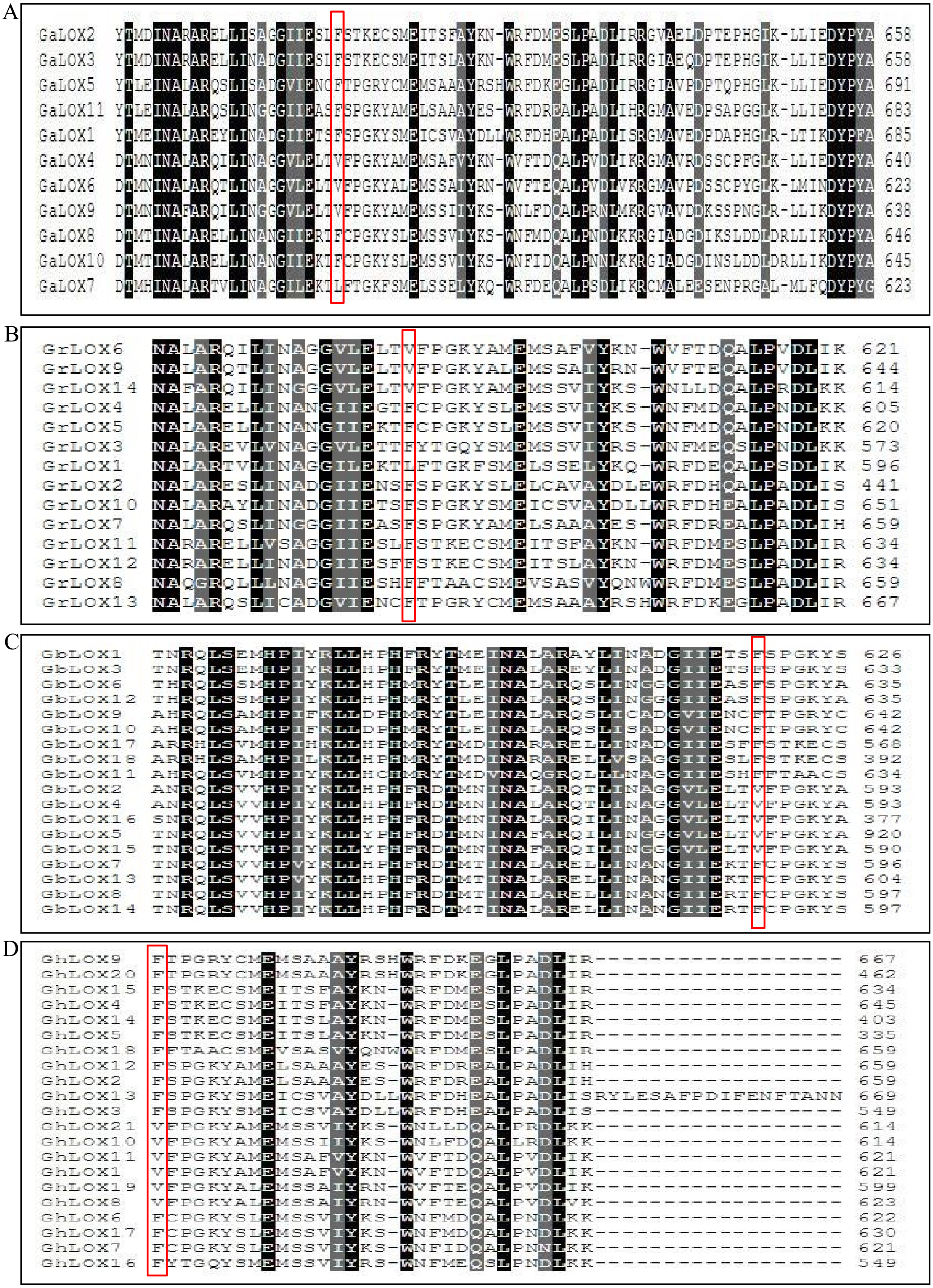
A
GaLOX2 YTMDINARARELLISAGGIIESLFSTKECSMEITSFAYKN-WRFDMESLPADLIRRGVAELDPTEPHGIK-LLIEDYPYA 658
GaLOX3 YTMDINARARELLINADGIIESLFSTKECSMEITSLAYKN-WRFDMESLPADLIRRGIAEQDPTEPHGIK-LLIEDYPYA 658
GaLOX5 YTLEINALARQSLISADGVIENCFTPGRYCMEMSAAAYRSHWRFDKEGLPADLIRRGIAVPDPTQPHGLK-LLIEDYPYA 691
GaLOX11 YTLEINALARQSLINGGGIIEASFSPGKYAMELSAAAYES-WRFDREALPADLIHRGMAVEDPSAPGGLK-LLIEDYPYA 683
GaLOX1 YTMEINALAREYLINADGIIETSFSPGKYSMEICSVAYDLLWRFDHEALPADLISRGMAVEDPDAPHGLR-LTIKDYPFA 685
GaLOX4 DTMNINALARQILINAGGVLELTVFPGKYAMEMSAFVYKN-WVFTDQALPVDLIKRGMAVRDSSCPFGLK-LLIEDYPYA 640
GaLOX6 DTMNINALARQTLINAGGVLELTVFPGKYALEMSSAIYRN-WVFTEQALPVDLVKRGMAVPDSSCPYGLK-LMINDYPYA 623
GaLOX9 DTMNINAFARQILINGGGVLELTVFPGKYAMEMSSIIYKS-WNLFDQALPRNLMKRGVAVDDKSSPNGLR-LLIKDYPYA 638
GaLOX8 DTMTINALARELLINANGIIERTFCPGKYSLEMSSVIYKS-WNFMDQALPNDLKKRGIADGDIKSLDDLDRLLIKDYPYA 646
GaLOX10 DTMTINALARELLINANGIIEKTFCPGKYSLEMSSVIYKS-WNFIDQALPNNLKKRGIADGDINSLDDLDRLLIKDYPYA 645
GaLOX7 DTMHINALARTVLINAGGILEKTLFTGKFSMELSSELYKQ-WRFDEQALPSDLIKRCMALEESENPRGAL-MLFQDYPYG 623
B
GrLOX6 NALARQILINAGGVLELTVFPGKYAMEMSAFVYKN-WVFTDQALPVDLIK 621
GrLOX9 NALARQTLINAGGVLELTVFPGKYALEMSSAIYRN-WVFTEQALPVDLIK 644
GrLOX14 NAFARQILINGGGVLELTVFPGKYAMEMSSVIYKS-WNLLDQALPRDLKK 614
GrLOX4 NALARELLINANGIIEGTFCPGKYSLEMSSVIYKS-WNFMDQALPNDLKK 605
GrLOX5 NALARELLINANGIIEKTFCPGKYSLEMSSVIYKS-WNFMDQALPNDLKK 620
GrLOX3 NALAREVLVNAGGVLETTFYTGQYSMEMSSVIYRS-WNFMEQSLPNDLKK 573
GrLOX1 NALARTVLINAGGILEKTLFTGKFSMELSSELYKQ-WRFDEQALPSDLIK 596
GrLOX2 NALARESLINADGIIENSFSPGKYSLELCAVAYDLEWRFDHQALPADLIS 441
GrLOX10 NALARAYLINADGIIETSFSPGKYSMEICSVAYDLLWRFDHEALPADLIS 651
GrLOX7 NALARQSLINGGGIIEASFSPGKYAMELSAAAYES-WRFDREALPADLIH 659
GrLOX11 NARARELLVSAGGIIESLFSTKECSMEITSFAYKN-WRFDMESLPADLIR 634
GrLOX12 NARARELLINADGIIESFFSTKECSMEITSLAYKN-WRFDMESLPADLIR 634
GrLOX8 NAQGRQLLLNAGGIIESHFFTAACSMEVSASVYQNWWRFDMESLPADLIR 659
GrLOX13 NALARQSLICADGVIENCFTPGRYCMEMSAAAYRSHWRFDKEGLPADLIR 667
C
GbLOX1 TNRQLSEMHPIYRLLHPHFRYTMEINALARAYLINADGIIETSFSPGKYS 626
GbLOX3 TNRQLSEMHPIYRLLHPHFRYTMEINALAREYLINADGIIETSFSPGKYS 633
GbLOX6 THRQLSSMHPIYKLLHPHMRYTLEINALARQSLINGGGIIEASFSPGKYA 635
GbLOX12 THRQLSSMHPIYKLLHPHMRYTLEINALARQSLINGGGIIEASFSPGKYA 635
GbLOX9 AHRQLSAMHPIFKLLDPHMRYTLEINALARQSLICADGVIENCFTPGRYC 642
GbLOX10 AHRQLSAMHPIFKLLDPHMRYTLEINALARQSLISADGVIENCFTPGRYC 642
GbLOX17 ARRHLSVMHPIHKLLHPHMRYTMDINARARELLINADGIIESFFSTKECS 568
GbLOX18 ARRHLSAMHPILKLLHPHMRYTMDINARARELLVSAGGIIESLFSTKECS 392
GbLOX11 AHRQLSVMHPIYKLLHCHMRYTMDVNAQGRQLLLNAGGIIESHFFTAACS 634
GbLOX2 ANRQLSVVHPIYKLLHPHFRDTMNINALARQTLINAGGVLELTVFPGKYA 593
GbLOX4 ANRQLSVVHPIYKLLHPHFRDTMNINALARQTLINAGGVLELTVFPGKYA 593
GbLOX16 SNRQLSVVHPIYKLLHPHFRDTMNINALARQILINAGGVLELTVFPGKYA 377
GbLOX5 TNRQLSVVHPIYKLLYPHFRDTMNINAFARQILINGGGVLELTVFPGKYA 920
GbLOX15 TNRQLSVVHPIYKLLYPHFRDTMNINAFARQILINGGGVLELTVFPGKYA 590
GbLOX7 TNRQLSVVHPVYKLLHPHFRDTMTINALARELLINANGIIEKTFCPGKYS 596
GbLOX13 TNRQLSVVHPVYKLLHPHFRDTMTINALARELLINANGIIEKTFCPGKYS 604
GbLOX8 TNRQLSVVHPIYKLLHPHFRDTMTINALARELLINANGIIERTFCPGKYS 597
GbLOX14 TNRQLSVVHPIYKLLHPHFRDTMTINALARELLINANGIIERTFCPGKYS 597
D
GhLOX9 FTPGRYCMEMSAAAYRSHWRFDKEGLPADLIR------------------ 667
GhLOX20 FTPGRYCMEMSAAAYRSHWRFDKEGLPADLIR------------------ 462
GhLOX15 FSTKECSMEITSFAYKN-WRFDMESLPADLIR------------------ 634
GhLOX4 FSTKECSMEITSFAYKN-WRFDMESLPADLIR------------------ 645
GhLOX14 FSTKECSMEITSLAYKN-WRFDMESLPADLIR------------------ 403
GhLOX5 FSTKECSMEITSLAYKN-WRFDMESLPADLIR------------------ 335
GhLOX18 FFTAACSMEVSASVYQNWWRFDMESLPADLIR------------------ 659
GhLOX12 FSPGKYAMELSAAAYES-WRFDREALPADLIH------------------ 659
GhLOX2 FSPGKYAMELSAAAYES-WRFDREALPADLIH------------------ 659
GhLOX13 FSPGKYSMEICSVAYDLLWRFDHEALPADLISRYLESAFPDIFENFTANN 669
GhLOX3 FSPGKYSMEICSVAYDLLWRFDHEALPADLIS------------------ 549
GhLOX21 VFPGKYAMEMSSVIYKS-WNLLDQALPRDLKK------------------ 614
GhLOX10 VFPGKYAMEMSSIIYKS-WNLFDQALLRDLKK------------------ 614
GhLOX11 VFPGKYAMEMSAFVYKN-WVFTDQALPVDLIK------------------ 621
GhLOX1 VFPGKYAMEMSAFVYKN-WVFTDQALPVDLIK------------------ 621
GhLOX19 VFPGKYALEMSSAIYRN-WVFTEQALPVDLIK------------------ 599
GhLOX8 VFPGKYALEMSSAIYRN-WVFTEQALPVDLVK------------------ 623
GhLOX6 FCPGKYSLEMSSVIYKS-WNFMDQALPNDLKK------------------ 622
GhLOX17 FCPGKYSLEMSSVIYKS-WNFMDQALPNDLKK------------------ 630
GhLOX7 FCPGKYSLEMSSVIYKS-WNFIDQALPNNLKK------------------ 621
GhLOX16 FYTGQYSMEMSSVIYRS-WNFMEQSLPNDLKK------------------ 549
