# Supplementary material for: Genome-wide identification of lipoxygenase gene family in cotton and functional characterization in response to abiotic stresses
Source: BMC Genomics. 2018 Aug 9;19:599. doi: 10.1186/s12864-018-4985-2 (PMC6085620; doi:10.1186/s12864-018-4985-2)
Supplement: Supplementary file 12 — Table S10. Primers used in this Study. (DOCX 16 kb) [file 12864_2018_4985_MOESM12_ESM.docx]

| GhUBQ7(S) | GAAGGCATTCCACCTGACCAAC |
| --- | --- |
| GhUBQ7(A) | CTTGACCTTCTTCTTCTTGTGCTTG |
| LX1802(S)  GhLOX4 | GCTCATTGAGCTTGATTCCGTC |
| LX1802(A)  GhLOX4 | CATTTGTTCCATCGCATCATATTC |
| LX1803(S)  GhLOX5 | GATGACGAAGAGTATTTAGGTGATCG |
| LX1803(A)  GhLOX5 | TGGCACCAGTAGTTGATAAGGC |
| LX704(S)  GhLOX18 | CCTTTATTCACTCCCAATGGCTAC |
| LX704(A)  GhLOX18 | CCCGAATCGTAAGGCAAATAGTT |
| LX504(S)  GhLOX9 | TCCTCTTTGTTAAACCTGTCTGGG |
| LX504(A)  GhLOX9 | GCAGTCACCTTAAACTCAACAGCT |
| LX1542(S)  GhLOX12 | GATTCGCCGCAAAACTTGGG |
| LX1542(A)  GhLOX12 | TCAAATGGAAACGCTGTTGGGTAT |
| LX683(S)  GhLOX13 | GTAAAACAGAAGGGTGACGACGT |
| LX683(A)  GhLOX13 | GATCAGTGAGACCATCAAGCACG |
| LX1863(S)  GhLOX6 | TCTTGAGGGGAGATGGGACTGG |
| LX1863(A)  GhLOX6 | AAGGATACTGAGCCAAACCTCCAA |
| LX2223(S)  GhLOX16 | CAGCAGCGTTGCGTGAATACAG |
| LX2223(A)  GhLOX16 | CACCCTTATCGGGATCACCCAA |
| LX888(S)  GhLOX10 | CTTCCTTCTGGACCTTTGCTTG |
| LX888(A)  GhLOX10 | TTGAATGACTTGAGGCACAGGA |
| LX294(S)  GhLOX1 | TATACCTCGGGCAGCGTGATAC |
| LX294(A)  GhLOX1 | AGCAATGTGTATGGCACCTTCA |
| LX2247(S)  GhLOX8 | CGTAACGACGGTGAGAGATTGA |
| LX2247(A)  GhLOX8 | ATTCTTGCAGCCTACTGATGATTA |

| **Gene name** | **Sense Primer** | **Anti-sense Primer** |
| --- | --- | --- |
| **LX683V**  GhLOX13 | CGGGGTACCGTAAAACAGAAGGGTGACGACGT | CGCGGATCCCGAGCACTGGTCTTTTCTTCTTGA |
| **LX1542V**  GhLOX12 | CGGGGTACCCGCAAGAAACCAATCCCGAC | CGCGGATCCTCAAATGGAAACGCTGTTGGGTAT |
| **LX1802V** | CGGGGTACCGCTCATTGAGCTTGATTCCGTC | CGCGGATCCGGCAGCCTTCTAGGGTGATG |
| **LX1863V** | CGGGGTACCTCTTGAGGGGAGATGGGACTGG | CGCGGATCCGATAAGATCAGTAGGAAGTGGGAGTC |
| **LX2223V** | GCGTGAGCTCGGTACCTCAATAAACCCAGCAAGAACACAA | GCCTCCATGGGGATCCCCATCTCCCCTCAAGACCTGTAAC |
